# Appraisal of Novel Oncological Therapies by the Scottish Medicines Consortium and the National Institute for Health and Care Excellence: A Comparative Study of Six Years of Data

**DOI:** 10.7759/cureus.50560

**Published:** 2023-12-15

**Authors:** Rory Taylor

**Affiliations:** 1 Internal Medicine, Queen Elizabeth University Hospital, Glasgow, GBR

**Keywords:** new drug approval, health technology assessment, oncology, pharmacoeconomics, the national institute for health and care excellence (nice), health policy and economics

## Abstract

Background and aims

Pharmacoeconomic assessment of novel oncological therapies is an increasingly important factor in determining patient access to therapies. Organisations such as the National Institute for Health and Care Excellence (NICE) in England and the Scottish Medicines Consortium (SMC) in Scotland assess medications for their cost-effectiveness through health technology assessments (HTA) and provide guidance on whether the public health service should fund a therapy. We assessed six years of data to determine if there were any differences in timescales and decisions between NICE and SMC for new oncological therapies.

Methods and results

Time (days) from marketing authorisation (MA) to publication of final HTA guidance was calculated for single technology appraisals published by NICE and SMC between January 1, 2017, and December 31, 2022, for oncological therapies. We assessed 161 HTAs by NICE and 148 HTAs by SMC published in the study period. The median time from MA to publication of HTA guidance was 291 days (IQR 222-406) for SMC and 257 days (IQR 167-448) for NICE (p=0.054). For solid organ cancer therapies, NICE was significantly faster in publishing guidance, with a median of 231.5 days (IQR 148-392.25), compared to SMC, which took 273 days (IQR 202-378) (p=0.039). Overall recommendation of technologies was similar between the SMC and NICE (90.5% and 89.4%, respectively), with discordance in a minority of cases (12.6%).

Conclusions

Recommendation rates for single HTAs are similar between NICE and SMC for oncological therapies with discordance in a minority of cases. The time from MA to publication of HTA guidance was similar overall, but NICE was faster in publishing HTA guidance for solid organ cancer indications. Differences in methodology and process between the two organisations, in particular the presence of the Cancer Drugs Fund in England, may explain this difference in publication times.

## Introduction

Novel oncological therapies are a major area of new drug development and comprise a substantial portion of new drug approvals in the United States and Europe [1,2], driven by modern advances in targeted molecular therapies [3]. However, newer anti-cancer therapies are increasingly expensive, and the therapeutic benefit provided is often modest in comparison to prices [4]. National regulatory agencies such as the Medicines and Healthcare Products Regulatory Agency (MHRA) in the United Kingdom (UK) assess whether medicines provide an appropriate benefit to the risk profile of patients to licence them for marketing but do not take economic aspects into account. Independent organisations such as the National Institute for Health and Care Excellence (NICE) in England and the Scottish Medicines Consortium (SMC) in Scotland are responsible for pharmacoeconomic appraisals to assess the cost-effectiveness of medications. These organisations publish guidance on whether the National Health Service (NHS), the public healthcare provider in the UK, should reimburse a treatment after assessing the clinical benefits of the treatment against the financial costs of the treatment. Cost-effectiveness is commonly defined through the quality-adjusted life year (QALY), equal to one year of life in perfect health [5], as a measure incorporating both lifespan and quality of life. The incremental cost per additional QALY added by a novel therapy compared to existing therapies is assessed as a metric of the health utility provided by a medicine. The SMC does not have a formal QALY threshold for funding, whilst NICE commonly utilises an incremental threshold of £20,000 to £30,000 per QALY added to guide funding decisions [6-8].

Although both organisations share the use of the QALY as a cost-effectiveness measure, they use different criteria, methodologies and implementation processes for appraising medicines. Despite this, the two organisations generally align on appraisal outcomes, although the SMC is noted to take longer to publish appraisals for oncology indications [9]. Whilst the health technology assessment (HTA) process provides another protection for the health service and taxpayers, the process can be lengthy and delay public access to medications. Common criticisms include ambiguity about decision-making, inappropriate funding decisions, lack of consistency, delays and lack of access for patients to treatments, particularly for cancer and orphan indications [10-14]. Healthcare providers in England have a statutory responsibility to make funding available for a medicine within 90 calendar days after guidance recommending its use is published by NICE. In Scotland, NHS providers are expected to reach a decision on the provision of an SMC-accepted medicine within 90 days of the SMC issuing advice to the health board that the medicine is recommended. Thus, the guidance of bodies such as NICE and SMC is integral to ensuring that novel oncological therapies are cost-effective and appropriate for funding, and their decisions and timelines can have significant impacts on the availability of therapeutic options for cancer patients in the UK.

## Materials and methods

Single health technology assessments (HTA) of oncological therapies published by NICE from January 1, 2017, to December 31, 2022, were identified on the NICE website. All appraisals of single technologies for oncology indications published by SMC between January 1, 2017, and December 31, 2022, were identified from published documents on the SMC website. The time from marketing authorisation (MA) until publication of HTA guidance was the primary outcome measure. The UK MA approval dates for the relevant indication or dates of label extension were obtained from the European Medicines Agency (EMA) or MHRA websites or the UK Summary of Product Characteristics for the product. If this data was not publicly available using the aforementioned sources, then it was obtained via an information request from the medical information department of the MA holder. Re-appraisals of technologies where HTA guidance was previously published were excluded. Assessments that were terminated due to non-submission by the MA holder were excluded. Differences in time from the date of the UK MA to publication of HTA between SMC and NICE were compared using a two-sided Mann-Whitney U test. P-values of <0.05 were considered statistically significant, and there was no control for multiplicity. Data collation and analysis were carried out in Microsoft Excel (Microsoft Corporation, Washington, USA) and Minitab version 21 (Minitab, LLC., Pennsylvania, USA).

## Results

**Figure 1:**
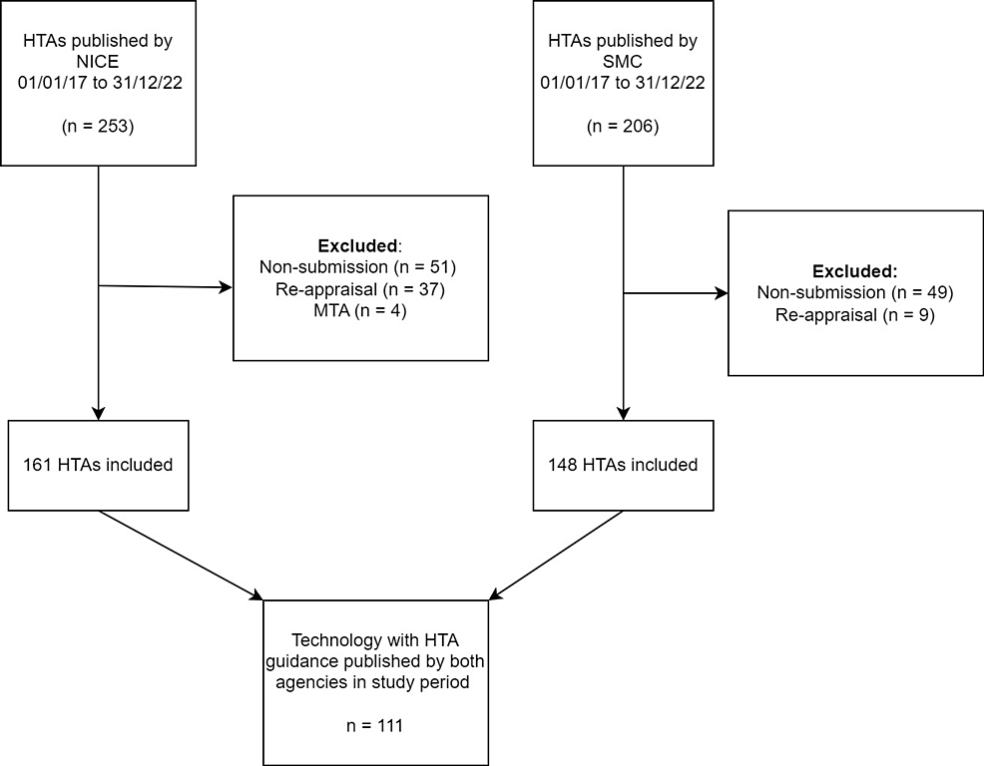
Flow diagram of the selection process Shows the flow diagram of the selection of published HTA guidance decisions with numbers and exclusions NICE: National Institute for Health and Care Excellence, SMC: Scottish Medicines Consortium, MTA: multiple technology assessment, HTAs: health technology assessments

Two hundred and six single HTAs were published by the SMC, and 253 were published by NICE between January 1, 2017, and December 31, 2022, for oncology therapies. Following exclusions, we included 148 HTAs for SMC and 161 HTAs for NICE, of which 111 technologies had HTA guidance published by both agencies during the study period. Figure 1 shows a flow diagram of the selection process.

Overall median time from MA to publication of guidance was not significantly different between organisations: 291 days (IQR 222-406) for SMC and 257 days (IQR 167-448) for NICE (p=0.054, not significant) (Table 1). A similar amount of technologies were recommended in guidance by SMC and NICE (90.5% and 89.4%, respectively), with a similar proportion recommended with restrictions on their use (29.7% SMC and 26.1% NICE). The majority of HTAs were for solid organ cancers (70% for both organisations) (Table 1). The median time from MA to publication of HTA guidance for solid organ cancers was significantly lower for NICE at 231.5 days (IQR 148-392.25) compared to SMC at 273 days (IQR 202-378) (p=0.039) (Table 1). There was no significant difference in time from MA to publication of HTA guidance for haematological malignancy between SMC and NICE (p=0.597) (Table 1). The most common tumour types for technologies were lung and breast for both organisations (SMC: breast 23 and lung 23 appraisals, NICE: breast 20 and lung 32 appraisals) (Table 2). The median time to publication of HTA guidance after MA was significantly shorter for solid organ cancer therapies than haematological malignancy for NICE (231.5 days (IQR 148-392.25) vs. 339 days (IQR 206-623), p=0.010) and SMC (273 days (IQR 202-378) vs. 327 days (IQR 258.5-780), p=0.012).

**Table 1:** Technologies assessed by either agency Shows the overview of HTA guidance published by either agency during the study period SMC: Scottish Medicines Consortium, NICE: National Institute for Health and Care Excellence, MA: marketing authorisation, IQR: interquartile range, HTAs: health technology assessments p-values of <0.05 are considered statistically significant

	SMC	NICE	p-value
Number of HTAs included	148	161	
Technology recommended (no restrictions) (%)	90/148 (60.8%)	102/161 (63.3%)	
Technology recommended (with restrictions) (%)	44/148 (29.7%)	42/161 (26.1%)	
Technology not recommended (%)	14/148 (9.5%)	17/161 (10.6%)	
Overall median time from MA to publication of guidance (days) (IQR)	291 (222-406)	257 (167-448)	0.054
Solid organ cancer HTAs (%)	105/148 (70.9%)	114/161 (70.8%)	
Solid organ cancer technology not recommended (%)	12/105 (11.4%)	16/114 (14.0%)	
Median time from MA to publication of guidance for solid organ cancer technology (days) (IQR)	273 (202-378)	231.5 (148-392.25)	0.039
Haematological malignancy HTAs (%)	43/148 (29.1%)	47/161 (29.2%)	
Haematological malignancy technology not recommended (%)	2/43 (4.7%)	1/47 (2.1%)	
Median time from MA to publication of guidance for haematological malignancy technology (days) (IQR)	327 (258.5-780)	339 (206-623)	0.597

**Table 2:** HTA guidance published by tumour type Shows the overview of HTA guidance published by tumour type SMC: Scottish Medicines Consortium, NICE: National Institute for Health and Care Excellence, GI: gastrointestinal

Tumour type	SMC	NICE
Myeloma (%)	10 (6.7%)	7 (4.3%)
Leukaemia (%)	15 (10.1%)	17 (10.6%)
Myeloproliferative disorder (%)	4 (2.7%)	4 (2.5%)
Lymphoma (%)	14 (9.5%)	20 (12.4%)
Lung (%)	23 (15.5%)	32 (19.9%)
Breast (%)	23 (15.5%)	20 (12.4%)
Prostate (%)	6 (4.1%)	8 (5.0%)
Hepatobiliary (%)	4 (2.7%)	5 (3.1%)
Upper GI (%)	5 (3.4%)	4 (2.5%)
Lower GI (%)	4 (2.7%)	3 (1.9%)
Melanoma (%)	5 (3.4%)	5 (3.1%)
Gynaecological	7 (4.7%)	6 (3.7%)
Urological (%)	17 (11.5%)	15 (9.3%)
Other (%)	11 (7.4%)	15 (9.3%)

There were 111 technologies with published guidance by both agencies between January 1, 2017, and December 31, 2022 (Table 3). For these technologies, the median time from MA to HTA publication date was significantly longer for SMC with 287 days (IQR 217-362) than NICE with 233 days (IQR 144-358) (p=0.005) (Table 3). A similar number of technologies were recommended in guidance for SMC and NICE (90.1% and 91%, respectively) (Table 3), and similar proportions were recommended with restrictions (28.8% SMC and 26.1% NICE). All technologies not recommended were for solid organ cancer. The median time from MA to publication of HTA guidance was significantly shorter for NICE than SMC for solid organ cancer (NICE 225 days (IQR 135-313), SMC 272 days (IQR 220.5-354.75) (p=0.006)) but not for haematological malignancy (NICE 293 days (IQR 179-489), SMC 318 days (266-400), p=0.338). There were 14 technologies assessed by both agencies where there was discordance in the final guidance recommendation (Table 4). Six technologies were rejected by NICE and approved by SMC, and eight technologies were rejected by SMC and approved by NICE (Table 4).

**Table 3:** Technologies assessed by both agencies Shows the overview of HTA guidance published by both agencies for the same agency during the study period SMC: Scottish Medicines Consortium, NICE: National Institute for Health and Care Excellence, MA: marketing authorisation, IQR: interquartile range, HTAs: health technology assessments p-values of <0.05 are considered statistically significant

	SMC	NICE	p-value
Number of HTAs	111	111	
Technology recommended (no restrictions) (%)	68/111 (61.3%)	72/111 (64.9%)	
Technology recommended (with restrictions) (%)	32/111 (28.8%)	29/111 (26.1%)	
Technology not recommended (%)	11/111 (9.9%)	10/111 (9.0%)	
Overall median time from MA to publication of guidance (days) (IQR)	287 (217-362)	233 (144-358)	0.005
Solid organ cancer HTAs (%)	82 (73.9%)	82 (73.9%)	
Solid organ cancer technology not recommended (%)	11/82 (13.4%)	9/82 (11.0%)	
Median time from MA to publication of guidance for solid organ cancer technology (days) (IQR)	272 (220.5-354.75)	225 (135-313)	0.006
Haematological malignancy HTAs (%)	29 (26.1%)	29 (26.1%)	
Haematological malignancy technology not recommended (%)	0 (0%)	0 (0%)	
Median time from MA to publication of guidance for haematological malignancy technology (days) (IQR)	318 (266-400)	293 (179-489)	0.338

**Table 4:** Technologies with discordance between agencies Shows technologies where NICE and SMC differed in overall recommendation guidance outcome (recommended vs. not recommended) CDF: Cancer Drugs Fund, identifier: identifier signature of HTA guidance document published on the agency website, SMC: Scottish Medicines Consortium, NICE: National Institute for Health and Care Excellence, GI: gastrointestinal

Technology	SMC guidance	SMC identifier	NICE guidance	NICE identifier	Tumour type
Alpelisib	Not recommended	SMC2481	Recommended (optimised)	NICE TA816	Breast
Ixekizumab	Not recommended	SMC2440	Recommended (optimised)	NICE TA718	Myeloma
Selpercatinib	Not recommended	SMC2371	Recommended (CDF)	NICE TA760	Lung
Olaparib	Recommended	SMC2366	Not recommended	NICE TA831	Prostate
Trifluridine/tipiracil (Lonsurf®)	Recommended (restricted)	SMC2329	Not recommended	NICE TA669	Upper GI
Pembrolizumab	Recommended (restricted)	SMC2247	Not recommended	NICE TA650	Urological
Abiraterone	Recommended	SMC2215	Not recommended	NICE TA721	Prostate
Atezolizumab	Not recommended	SMC2208	Recommended	NICE TA584	Lung
Cabozantinib	Not recommended	SMC2316	Recommended	NICE TA542	Urological
Pembrolizumab	Not recommended	1339/18	Recommended (CDF)	NICE TA522	Urological
Obinutuzumab	Not recommended	SMC2015	Recommended	NICE TA513	Lymphoma
Regorafenib	Recommended	1316/18	Not recommended	NICE TA514	Hepatobiliary
Atezolizumab	Not recommended	1297/18	Recommended	NICE TA525	Urological
Osimertinib	Recommended (restricted)	SMC2382	Not recommended	NICE TA621	Lung

## Discussion

In this study, we assessed 161 single HTAs published by NICE and 148 by SMC for oncological therapies. A similar proportion of technologies were recommended for use by both agencies (SMC 90.5%, NICE 89.4%). The number of technologies that were recommended with restrictions on their use was also similar between agencies. An overall recommendation rate of 85% is reported by NICE for data from 2000 to 2023 [15], although this also covers appraisals for non-oncology therapies. A 2012 study by Ford et al. reported an 80% recommendation rate for NICE and a 71.4% recommendation for SMC for cancer therapies [9]. This study reported a median time from MA to publication of single HTA guidance for cancer drugs of 25.2 months for NICE and 8.0 months for SMC. That study looked at data from different decades (the 2000s vs. 2010s/2020s), where NICE and SMC processes were different and the method of data extraction for SMC was different (from annual appraisal summary documents rather than single published guidance documents), which may explain the discrepancies seen. More recent studies looking at HTA final guidance documents between 2014 and 2016 for oncology therapies found recommendation rates of 79% for NICE and 75% for SMC [16,17].

In our study, there was disagreement between SMC and NICE on recommendation or non-recommendation for a minority of technologies (12.6%). This is consistent with previous work that has shown a high degree of agreement between SMC and NICE for cancer therapies compared to other European countries [18,19]. We found that NICE published faster guidance for solid organ therapies but not haematological malignancy therapies than SMC, although the reasons for this are unclear. NICE was significantly faster at publishing guidance than SMC for technologies that were assessed by both agencies during the study period, which was driven by faster times for solid organ cancer therapies, with no difference in timelines seen in therapies for haematological malignancy. This likely reflects the fact that technologies appraised by both agencies in the study period were likely to be completely novel therapies in high-impact areas or areas of unmet need, which may have led to an acceleration of timelines for both agencies, although this appears to have been more pronounced for NICE than SMC.

The Cancer Drugs Fund (CDF) was set up in England in 2010 to improve access to novel cancer therapies. The CDF provided reimbursement for therapies that were pending appraisal by NICE or have been rejected by NICE due to a lack of cost-effectiveness or immature data for health economic models [20]. In 2016, the CDF was reformed due to multiple consecutive years of spending outside of its allotted budget and re-aligned with NICE [20,21]. These reforms included changes to the NICE appraisal process, such that the process began earlier, with initial submissions and reviews occurring prior to the drug receiving MA approval, thereby reducing delays to patient access after MA approval by the MHRA [20]. Previous work has indicated that 2014 reforms at the SMC for the approval of end-of-life and orphan indications had led to increased access to therapies for advanced cancer, although this only addressed approval rates and not timelines to approval [16,22]. The difference in processes between NICE and the CDF may provide some explanation as to why NICE continues to publish oncology HTA guidance faster than the SMC. Other factors that can account for discrepancies between the two agencies that have previously been published in the literature include the method of dealing with uncertainties about cost-effectiveness, comparator choice, clinical benefits [23,24], negotiation of patient access schemes and market entry agreements [18], consideration of indirect benefits of treatment and the innovative nature of treatments [25,26]. The increased recommendation rate observed in our studies compared to previous work likely reflects the ongoing consequences of reforms made in the 2010s at both organisations and further refinements in the approach of manufacturers and agencies to increase access to oncological therapies.

Delays in publication of HTA guidance can occur for a variety of reasons. The type of technology appraised is relevant, as both therapies for oncology and orphan status are known to extend the appraisal process duration [27,28]. Initial draft guidance by NICE is negative in 60% of cases [29] and leads to significant delays in the issuance of final guidance [28]. The manufacturer's response to the draft guidance is also important in terms of the final outcome. In one study, 38% of preliminary negative decisions could receive recommendations in final guidance after the introduction or enhancement of a patient access scheme discount, which would be at the discretion of the manufacturer [28]. Drugs for advanced cancers are also known to require more committee meetings prior to the decision and a longer time from the first meeting until publication of final guidance than non-oncology therapies for both SMC and NICE [9,28].

Limitations

This study has a number of limitations. We assessed only the time until publication of final guidance and did not take into account where draft guidance rejecting the drug was issued before the company resubmitted with final approval (which may come with substantial time delays). Additionally, the time to publication of results may be affected due to a lack of, or delay in, submission of data by the MA holder as well as delays in the SMC or NICE process, which our study does not distinguish between. We also assumed that the decision-making processes of NICE and SMC are independent; however, companies may change the contents of the submitted dossier, including their proposed pharmacoeconomic models, on the basis of feedback or interactions with the other agency. Furthermore, the initial models submitted by the manufacturer to the respective agencies may be different, which can lead to increased meetings and time until publication of the guidance or differences in the outcome of the HTA process. Finally, we only looked at decisions within the UK, although evidence suggests there is significant variation in HTA assessment and recommendations between G7 countries [30].

## Conclusions

Recommendation rates are similar for single health technology assessments of oncological therapies for both NICE and SMC (89.4% and 90.5%), with only a minority of therapies (12.6%) having discordance in recommendation outcomes between the two organisations. NICE published guidance significantly faster than the SMC for solid organ cancer therapies, but timelines were similar for haematological malignancies. This is most likely due to differences in process and methodology between the two agencies, in particular the role of the CDF in England.
